# Estimating the effect of timing of earned income tax credit refunds on perinatal outcomes: a quasi-experimental study of California births

**DOI:** 10.1186/s12889-023-16920-0

**Published:** 2023-11-07

**Authors:** Deborah Karasek, Akansha Batra, Rebecca J. Baer, Brittany D. Chambers Butcher, Sky Feuer, Jonathan D. Fuchs, Miriam Kuppermann, Anu Manchikanti Gomez, Aric A. Prather, Matt Pantell, Elizabeth Rogers, Jonathan M. Snowden, Jacqueline Torres, Larry Rand, Laura Jelliffe-Pawlowski, Rita Hamad

**Affiliations:** 1 School of Public Health, Oregon Health & Science University – Portland State University, Portland, USA; 2 Department of Obstetrics, Gynecology, and Reproductive Sciences, University of California, San Francisco, San Francisco, USA; 3 California Preterm Birth Initiative, University of California, San Francisco, San Francisco, USA; 4 Department of Epidemiology and Biostatistics, University of California, San Francisco, San Francisco, USA; 5 Department of Pediatrics, University of California, San Diego, San Diego, USA; 6 Department of Human Ecology, University of California, Davis, Davis, USA; 7 Department of Public Health, Population Health Division, San Francisco, USA; 8 Sexual Health and Reproductive Equity Program, School of Social Welfare, University of California, Berkeley, Berkeley, USA; 9 Department of Psychiatry, University of California, San Francisco, San Francisco, USA; 10 Department of Pediatrics, University of California, San Francisco, San Francisco, USA; 11 Department of Family and Community Medicine, Philip R. Lee Institute for Health Policy Studies, University of California, San Francisco, San Francisco, USA; 12 Department of Social and Behavioral Sciences, Harvard School of Public Health, Harvard University, Cambridge, USA

**Keywords:** Pregnancy outcomes, Preterm birth, Policy evaluation, Income effects, Maternal health, Gestational diabetes, Gestational hypertension

## Abstract

**Background:**

The largest poverty alleviation program in the US is the earned income tax credit (EITC), providing $60 billion to over 25 million families annually. While research has shown positive impacts of EITC receipt in pregnancy, there is little evidence on whether the timing of receipt may lead to differences in pregnancy outcomes. We used a quasi-experimental difference-in-differences design, taking advantage of EITC tax disbursement each spring to examine whether trimester of receipt was associated with perinatal outcomes.

**Methods:**

We conducted a difference-in-differences analysis of California linked birth certificate and hospital discharge records. The sample was drawn from the linked CA birth certificate and discharge records from 2007–2012 (*N* = 2,740,707). To predict eligibility, we created a probabilistic algorithm in the Panel Study of Income Dynamics and applied it to the CA data. Primary outcome measures included preterm birth, small-for-gestational age (SGA), gestational diabetes, and gestational hypertension/preeclampsia.

**Results:**

Eligibility for EITC receipt during the third trimester was associated with a lower risk of preterm birth compared with preconception. Eligibility for receipt in the preconception period resulted in improved gestational hypertension and SGA.

**Conclusion:**

This analysis offers a novel method to impute EITC eligibility using a probabilistic algorithm in a data set with richer sociodemographic information relative to the clinical and administrative data sets from which outcomes are drawn. These results could be used to determine the optimal intervention time point for future income supplementation policies. Future work should examine frequent income supplementation such as the minimum wage or basic income programs.

**Supplementary Information:**

The online version contains supplementary material available at 10.1186/s12889-023-16920-0.

## Background

Socioeconomic status during pregnancy is an increasingly recognized determinant of adverse birth outcomes and later life health for both the birthing person and child [1, 2]. Research has consistently documented a relationship between living in poverty and preterm birth and low birthweight (LBW) [1, 3, 4]. On average, U.S families see a 10 percent decline and single mothers a 41 percent decline in income during pregnancy [5]. There is growing consensus around the need to address social and structural factors that drive adverse birth outcomes in the U.S [2, 6].

There is limited work that examines the effects of poverty reduction interventions on birth outcomes, and little evidence on whether the timing of such interventions may lead to differences in health outcomes. In the U.S., the earned income tax credit (EITC) is the largest poverty alleviation program, providing income supplementation to working families in the form of tax rebate contingent their employment. The size of the credit increases with increasing earned income, eventually plateauing followed by a phase-out of benefits [7]. Initiated in 1975, the program was expanded in 1993, creating variation in the size of the tax credit received by recipients. EITC policies receive bipartisan support, with over half of all states offer differing amounts of supplemental EITC through policy expansions [8]. In 2018, over 25 million beneficiaries received US$63 billion, averaging about US$2,500 per family [9], and half of recipients were single mothers [10]. Studies have shown that EITC has lifted millions of families out of poverty, increased labor force participation, housing access, and improved health [11–15], however effects on fertility and marriage are less clear [16].

The EITC may impact birth outcomes of interest through several hypothesized pathways, including reductions in stress associated with financial insecurity, or increases in material resources such as housing, nutrition, healthcare access, and transportation.

A handful of studies have evaluated the effects of the EITC on birth outcomes, most demonstrating improvements [17–20]. The majority, however, relied on historical data prior to 2000 and examined only birthweight. Growing income inequality and a weaker safety net make more contemporary evaluations of the EITC increasingly important for population health [21]. While studies have found that state EITC programs (which provide a small supplement to the federal EITC) improved birthweight and preterm birth rates, in particular for Black pregnant women and people [20, 22–24], only one study to our knowledge examined whether the trimester of EITC income disbursement differentially affected outcomes, with a small sample and null results [17]. A study of transfers among Special Supplemental Nutrition Program for Women, Infants, and Children (WIC) clients suggested strongest improvements in birth outcomes for receipt in the third trimester [25].

We estimated the effects of the trimester of eligibility for EITC income receipt on perinatal outcomes, using data from more than 2 million births in California. Leveraging the fact that EITC income is received after taxes are filed in the spring and that timing of receipt is unlikely to be associated with individual characteristics, we used a quasi-experimental difference-in-differences (DID) design. DID compares perinatal outcomes 1) among pregnant people who receive EITC during first, second, and third trimester compared to preconception and 2) and between pregnant people who were likely to receive EITC and those who were not. This second comparison “differences out” any seasonal variation in birth outcomes by trimester among non-EITC eligible pregnant people. Given racial inequities in birth outcomes, we also conducted subgroup analyses by race/ethnicity.

## Methods

### Data

The sample was drawn from all live births between 2007 and 2012 using the California Office of State Health Planning and Development database, containing birth and death certificates linked to hospital discharge records. It included information on maternal and infant characteristics, discharge diagnoses, and procedures recorded as early as one year prior to birth for the birthing person and as late as one-year post-birth for the birthing person and infant. The data set was restricted to singleton live-born infants with a gestational age between 26 and 42 weeks at birth, to allow exposure to all trimesters of pregnancy. The final sample size was 2,740,707 births to 2,321,353 people. Prior work has validated the use of outcome and covariate data from birth certificates and hospital discharge records [26–28]. The study was approved by the Committee for the Protection of Human Subjects (CPHS) Institutional Review Board (IRB) within the Health and Human Services Agency of California (protocol #12–09-0702 l). Consent was waived by the CPHS IRB as the study uses publicly available administrative data.

### Variables

#### Exposure

The primary exposure was eligibility for an EITC refund in the preconception period, compared to the first, second, or third trimester of pregnancy. As with most administrative data, the records do not contain information on EITC receipt. We therefore used a probabilistic algorithm to create a proxy measure of EITC eligibility using the 2001–2015 waves of the Panel Study of Income Dynamics (PSID, *N* = 3,672 people who reported giving birth in the past year). PSID includes self-reported information on income, household size, marital status, and age, which we used to calculate each individual’s EITC eligibility using Internal Revenue Service (IRS) formulas implemented as part of the *taxsim* package for Stata [29]. PSID has been used to examine the effect of EITC on perinatal and child health outcomes [20, 30]. We predicted individual probabilities of EITC eligibility using variables contained in both datasets: mothers’ receipt of WIC during pregnancy, race/ethnicity, parity, education, and age at birth. The inclusion of race/ethnicity reflects the overrepresentation of Black and Latine people among EITC recipients[31], a result of racist structures that have produced inequities in wealth and income [32]. In keeping with previous investigations [33], after generating weights for each variable from PSID, we applied them to the California birth data to predict likelihood of EITC eligibility. Predicted probabilities of 0.5 or greater were classified as EITC-eligible. Of note, this is an alternative to previous studies of the EITC that have used low educational attainment, Medicaid coverage, or state EITC policy passage as proxy measures for EITC eligibility [19, 23, 34, 35].

Prior work has shown that 80 percent of eligible individuals actually receive the EITC [36]. Therefore, we assumed that recipients received EITC refunds if they were imputed as eligible and refer to eligibility of income receipt as EITC receipt. While this results in some degree of misclassification, it is analogous to an intent-to-treat approach.

**Fig. 1:**
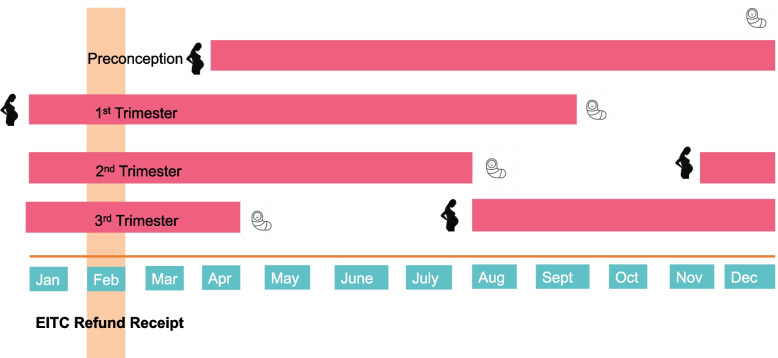
Schematic of classification of pregnancies into trimester of earned income tax credit (EITC) refund receipt, for four sample 9-month gestations. EITC eligibility determined by probabilistic using the 2001–2015 waves of the Panel Study of Income Dynamics. Pregnancies were classified as being exposed to the EITC in the preconception period (i.e., during the three months before conception), first trimester, second trimester, or third trimester, based on used date of birth and gestational age at birth

Our study capitalizes on the fact that about half of EITC refunds are issued in February, because they are disbursed as a tax refund [15, 37]. We used month of birth and gestational age at birth, obtained by best obstetric estimate, to calculate the trimester of exposure to the EITC refund (Fig. 1). Pregnancies were classified as being exposed to the EITC in the three months before conception, first trimester, second trimester, or third trimester. Other studies have leveraged this seasonal variation in EITC receipt to examine the short-term effects of income on adult and child health outcomes [14, 15, 17, 38].

#### Outcome

Infant outcomes included variables abstracted from birth certificates and hospital discharge records, which are known to be associated with maternal stress or material resources during pregnancy: preterm birth (i.e., birth before 37 weeks gestation) and small for gestational age (SGA, i.e. born at < 10^th^ percentile of birthweight for gestational age and sex). We examined two maternal outcomes from hospital discharge records: whether the mother developed gestational diabetes mellitus (GDM) in pregnancy and whether she developed gestational hypertension or preeclampsia. Pregnant people with preexisting diabetes were excluded from the GDM analysis. Pregnant people with preexisting hypertension that did not develop preeclampsia were coded as part of the non-outcome group.

#### Covariates

Covariates included sociodemographic variables on the birth certificate: mother’s age, age-squared, race/ethnicity (non-Latina white, non-Latina Black, Latina, Asian/Pacific Islander, other race/ethnicity), education (less than high school, high school, more than high school), Medicaid insurance, parity, infant sex assigned at birth, and indicator variables for year of birth.

### Patient and public involvement

Patients were not directly involved in this study, as it uses birth records and hospital discharge data.

#### Analysis

We tabulated sample characteristics among those classified as EITC-eligible and ineligible. We then used a difference-in-differences (DID) approach [39–41] to estimate the association of EITC eligibility with likelihood of each outcome, depending on trimester of receipt. We compared outcomes among those exposed to the EITC during the first, second, or third trimester to those exposed in the preconception period. DID then differenced out trends in outcomes among the EITC-ineligible group, accounting for trends in the outcomes due to seasonal differences shared in both EITC-eligible and EITC-ineligible groups.

DID models included an interaction term between a binary variable for EITC eligibility (i.e., EITC probability of 0.5 or greater) and trimester of exposure. Both continuous and binary outcomes were modeled using linear regression models. This is standard for DID analyses, because of differences in the interpretation of interaction terms in non-linear models [42, 43]. Coefficients for binary outcomes can be interpreted as the percent change in risk. We conducted a complete-case analysis. The specification of our model can be found in the *supplement.*

We ran our primary models stratified by race/ethnicity, to estimate any differences in the associations.

#### Sensitivity analyses

We conducted several sensitivity analyses to test differences in the EITC receipt probability threshold and to address potential bias in the timing of the cohort selection (see Supplementary Material).

## Results

Nearly 45 percent of the births were classified as EITC-eligible (*n* = 1,256,199) (Table 1), similar to the proportion of children in California overall that receive EITC (35 percent) [44]. EITC-eligible people were more likely to experience preterm birth and develop GDM, but were less likely to experience SGA, gestational hypertension, and preeclampsia (Table 2). Over 97% of the potential EITC beneficiaries were to people with prior births, mirroring prior reports of EITC recipients [45].

**Table 1:** Sociodemographic characteristics by EITC eligibility status

	EITC Ineligible (*n* = 1,484,508)		EITC Eligible (*n* = 1,256,199)		Total (*n* = 2,740,707)	
	No.	%	No.	%	No	%
Race						
Non-Latine White	631,398	42.53	122,427	9.75	753,825	27.5
Non-Latine Black	46,857	3.16	106,036	8.44	152,893	5.58
Latine	583,177	39.28	816,686	65.01	1,399,863	51.08
Non-Latine Asian/PI	194,912	13.13	172,723	13.75	367,635	13.41
Non-Latine Other	28,164	1.9	38,327	3.05	66,491	2.43
Parity						
1	1,050,115	70.74	31,649	2.52	1,081,764	39.47
2	263,037	17.72	599,750	47.74	862,787	31.48
3 +	171,356	11.54	624,800	49.74	796,156	29.05
Education						
> 12 years	1,002,588	67.54	320,821	25.54	1,323,409	48.29
≤ 12 years	481,920	32.46	935,378	74.46	1,417,298	51.71
Received WIC^a^	735,534	49.55	739,616	58.88	1,475,150	53.82
Insurance coverage						
Private or other	882,669	59.46	523,860	41.7	1,406,529	51.32
Medicaid	601,839	40.54	732,339	58.3	1,334,178	48.68
Age at delivery, years mean (SD)]	27.3		29.4		28.3	
Infant female	722,939	48.7	613,486	48.84	1,336,425	48.76
Year of birth						
2007	258,151	17.39	238,565	18.99	496,716	18.12
2008	255,096	17.18	228,912	18.22	484,008	17.66
2009	245,233	16.52	210,788	16.78	456,021	16.64
2010	242,636	16.34	198,958	15.84	441,594	16.11
2011	241,407	16.26	192,197	15.3	433,604	15.82
2012	241,985	16.30	186,779	14.87	428,764	15.64

^a^Mothers’ receipt of Special Supplemental Nutrition Program for Women, Infants, and Children (WIC) benefits during pregnancy

**Table 2:** Infant and maternal health characteristics by EITC eligibility status

	EITC Ineligible (*n* = 1,484,508)		EITC Eligible (*n* = 1,256,119)		Total	
	N	%	N	%	N	%
Preterm birth	95,314	6.42	90,815	7.23	186,129	6.79
Small for gestational age	138,324	9.32	95,837	7.63	234,161	8.54
Gestational diabetes	104,024	7.05	127,490	10.25	231,514	8.52
Preeclampsia or hypertension	98,756	6.68	53,277	4.26	152,033	5.57

### Association of trimester of EITC receipt

**Fig. 2:**
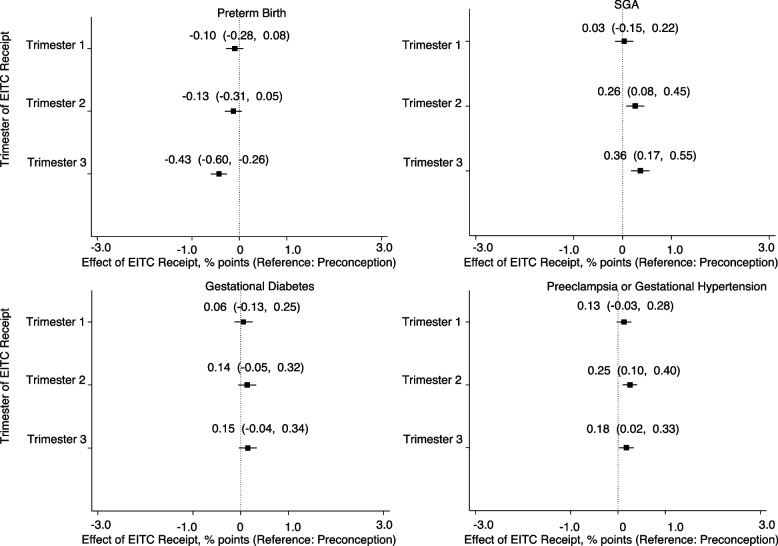
Associations of trimester of EITC receipt with perinatal outcomes. Coefficients represent the interaction term between EITC receipt in the first, second or third trimester compared to preconception. Coefficients for binary outcomes were multiplied by 100 and therefore represent a change in percentage points. Values in parentheses represent 95% confidence intervals. Analyses involved multivariable linear regression models (i.e., linear probability models for binary outcomes) with robust standard errors clustered by mother. Covariates included mother’s race/ethnicity, education, insurance, age, parity and infant’s sex and year of birth

Receipt of EITC refund in the third trimester was associated with a reduction in the likelihood of preterm birth compared with receipt in the preconception period (-0.43 percentage points, 95% CI: -0.60, -0.26) (Fig. 2), and there was no difference for EITC receipt in the first or second trimester. This represents at 6.3 percent reduction relative to a base PTB rate of 6.8 percent. EITC receipt in the second or third trimester was associated with increase in SGA birth [(0.26 percentage points, 95% CI: 0.08, 0.45) and (0.36 percentage points, 95% CI: 0.17, 0.55), respectively] compared with receipt during preconception. This represents a 3.0 percent and 4.2 percent increase from a base rate of 8.5 percent. Second or third trimester of EITC receipt was associated with a small increase in gestational hypertension or preeclampsia (0.25 percentage points, 95% CI: 0.10, 0.40). EITC receipt in any trimester compared to preconception was not associated with an increased risk of GDM.

### Differences by race/ethnicity

**Fig. 3:**
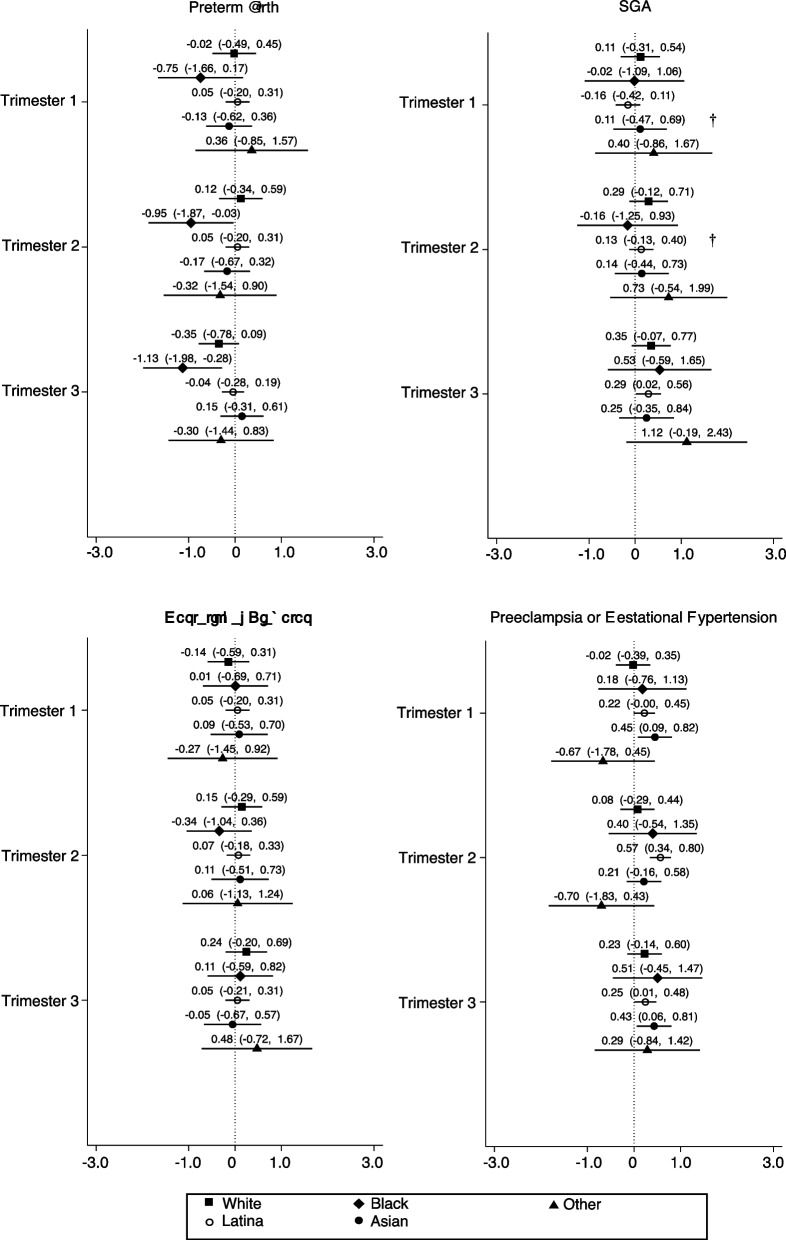
Associations of trimester of EITC receipt with perinatal outcomes by maternal race/ethnicity. Coefficients represent the effect of EITC receipt in the first, second, or third trimester compared with receipt during the preconception period. Coefficients for binary outcomes were multiplied by 100 and therefore represent a change in percentage points. Values in parentheses represent 95% confidence intervals. Analyses involved multivariable linear regression models with robust standard errors clustered by mother and stratified by maternal race/ethnicity. Covariates included education, insurance, age, age-squared, parity and infant’s sex, and year of birth. †: interaction term with *p* < 0.10

Stratified models revealed differences in the association of the trimester of EITC receipt with birth outcomes by maternal race/ethnicity (Fig. 3), although no group consistently varied from the overall estimates across all outcomes. For white pregnant people, there were no differences in preterm birth between EITC receipt in the preconception period and any other trimester. For Black pregnant people, refund receipt in the second trimester also conferred a protective association with likelihood of preterm birth. Confidence intervals were wide and cross the null for nearly every race/ethnicity for GDM, SGA, preeclampsia, or gestational hypertension, apart from a slight increase in the association with SGA for Latine pregnant people exposed in the third trimester. Preeclampsia, or gestational hypertension risk increased in second and third trimester for Latine pregnant people, and first trimester for Asian/Pacific Islander pregnant people, but not for other racial/ethnic groups.

### Sensitivity analyses

Analyses in which EITC eligibility was determined based on a probability threshold of 0.6 yielded similar results to the main analysis (eTable 1), although the effects for hypertension were no longer significant. When further restricting the probability threshold to 0.07, we observed stronger protective effects for PTB in all trimesters compared to preconception. Compared with preconception, EITC receipt in the second trimester was no longer associated with changes in gestational hypertension, however receipt in the third trimester showed a protective effect for gestational hypertension (eTable 2) Truncating the study population to conception cohorts that had full risk for all gestational age outcomes resulted in significant protective effects for the first and second trimester as well as the third for PTB and similar results for other outcomes (eTable 3).

## Discussion

In this study, we leveraged the timing of EITC disbursement in February of each year as a natural experiment to examine whether there is a sensitive period in pregnancy in which receipt of additional income could differentially affect perinatal outcomes. EITC receipt in the third trimester of pregnancy was associated with a 6 percent reduction in preterm birth relative to receipt during the preconception period. In contrast, receipt in the second or third trimester was associated with a 3 percent and 4 percent increase in SGA risk compared to receipt during preconception. There were no differences in maternal gestational diabetes based on trimester of EITC receipt, and a reverse pattern for was noted gestational hypertension and preeclampsia, in which receipt in the second and third trimesters was associated with increased risk compared to preconception.

EITC refunds may impact the outcomes of interest through several hypothesized pathways, including reductions in stress associated with financial insecurity, or increases in material resources such as housing, nutrition, and transportation. Our findings suggest that there may be different windows of opportunity for these mechanisms to impact health, depending on the outcome. For example, the stronger association for preterm birth for income received during the third trimester suggest that interventions to reduce stress and improve material resources in the period immediately before birth may have a substantial impact on outcomes related to gestational age at birth. While assessing a different exposure, this work is consistent with seminal work from the Dutch Famine Birth Cohort Study finding that exposure to famine in the third trimester resulted in lower birthweights compared with exposure in earlier trimesters [46]. Meanwhile, our findings imply that intervening on gestational hypertension and preeclampsia, which may be diagnosed later in pregnancy, may require investments in the preconception period, perhaps because the pathways that influence disease are initiated prior to or early in pregnancy. The lack of findings for GDM is consistent with a prior study that found that improvements to nutrition during pregnancy did not change the risk of gestational diabetes [47]. Findings of increased SGA for receipt in the later trimesters compared to preconception, could also indicate that receipt of income in the period before pregnancy buffers against growth restriction in pregnancy. This relationship could also be due to decreased fetal loss during early pregnancy, in accordance with the Wells hypothesis that posits that loss of small fetuses varies with maternal experience of stress [48].

Racial/ethnic inequities in birth outcomes led us to test for heterogeneity by race/ethnicity. We found that reductions in preterm birth among those receiving the EITC in the third trimester were greatest among Black pregnant people, the racialized group at highest risk for preterm birth. This result mirrors findings of stronger birthweight improvements for Black pregnant people following food stamp receipt in the third trimester [49]. Wealth plays an important role in weathering income volatility that may occur during pregnancy, and racial wealth disparities in the U.S. result from historical structural practices of racial exclusion [32]. Racial inequalities in income and wealth have been identified as one manifestation of structural racism [50, 51]. It may be that Black pregnant people experience greater benefits from income in later pregnancy due to lower levels of wealth to buffer against financial stress and volatility. These findings are consistent with recent studies that have shown state and federal EITC are associated with greater benefits on birth outcomes among Black pregnant people [24]. They contrast with one prior study finding greater risk of very LBW among Black pregnant people in California [34], although that study relied on historical data prior to 2000 and used educational attainment and Medicaid coverage as proxies for EITC receipt, which may have selected a different exposed population.

Our study has several strengths. First, we used a quasi-experimental design and a large population of births to provide rigorous evidence of the association of timing of income supplementation on a range of birth outcomes. Second, we used a novel method to impute EITC eligibility using a probabilistic algorithm in a data set with richer sociodemographic information relative to the clinical and administrative data sets from which outcomes are drawn.

This study also has several limitations. First, the linked birth certificate data do not include information on EITC receipt, EITC eligibility, or income, increasing likelihood of exposure misclassification. This is a common drawback in survey and administrative data, and our use of a probabilistic algorithm is an improvement over prior studies that used educational attainment or state EITC policies as crude proxies for eligibility [19, 23, 34, 35]. Future studies should attempt to link administrative income and tax data to the birth files. Of note, individuals and families that do not file a tax return due to income below the IRS filing threshold are likely to miss out on the EITC benefit, despite being eligible [52]. Second, DID analysis relies on the parallel trends assumption that the rate of change in the outcomes among the EITC-eligible and ineligible groups would have been the same in the absence of the EITC. Trends in monthly birth outcomes by EITC receipt and trimester reveal similar patterns, providing some assurance of the assumption. Therefore, this study relies on the assumption that no other events in February would have differentially influenced outcomes among the EITC-eligible and ineligible groups. Third, our study was conducted in California, and results may not generalize to other states. While our dataset is older, our study period from 2007–2012 includes a period prior of the 2015 California EITC benefit initiation; therefore the effects for federal EITC policy may be more generalizable to other states. Fourth, assignment of pregnancies to trimester of receipt may have induced different risk probabilities for preterm birth, given that gestations reaching the third trimester are no longer at risk of a previable birth. To address this, we limited the sample to births after 26 weeks. The DID design also addresses this concern but comparing the third trimester effects across EITC eligible and ineligible individuals. Finally, selection of February as the month of EITC receipt may result in misclassification of people who receive the rebate in the subsequent months. Measurement error may also have resulted from assignment of trimester of pregnancy based on month of conception and birth, and lack of specificity around date of EITC receipt. This assumption is consistent with previous studies in this area [14, 15].

Importantly this analysis is not directly testing whether EITC receipt in pregnancy impacts birth outcomes, which has been demonstrated in other analyses [17, 18, 38], but when in pregnancy it is most beneficial. The findings of increased gestational hypertension and preeclampsia later in pregnancy are relative to receipt in the preconception period and should not be interpreted as comparing EITC receipt to non-receipt. The length of pregnancy, combined with cyclical nature of tax season prevented us from looking at the postpartum period as a negative control. Selection of the postpartum period would also overlap with EITC receipt preconception in the prior year.

In this study, we provide some of the first evidence on how timing of receipt of income during pregnancy affects a range of perinatal health outcomes. These findings have important implications for understanding the effects of economic policy on early life health. These findings for EITC policies extend to international contexts, where cash transfer programs have been found to have small, but potentially important population-level effects on birthweight [53]. As the results do not point to a particular window in pregnancy for influencing every perinatal outcome, we believe further work to zero in on mechanism will be important. However, the findings provide evidence for the design of policies and programs that aim to buffer the impacts of economic insecurity in pregnancy and can be incorporated into cost-effectiveness analyses of economic interventions. Future work could examine other policies that deliver income through other mechanisms, such as minimum wage increases or guaranteed income, to explore consistency with these results.

### Supplementary Information

**Additional file 1:** **Supplementary Online Content**, **Supplementary Tables 1, 2 **and** 3.**

## Data Availability

The data that support the findings of this study are available from the California Health and Human Services Agency but restrictions apply to the availability of these data, which were used under license for the current study, and so are not publicly available. Data are however available upon reasonable request from the California Health and Human Services Agency and with permission of the California Health and Human Services Agency. For more information contact the Health Information and Research Section (HIRS) at HIRS@cdph.ca.gov.

## Abbreviations

EITC: Earned income tax credit
PTB: Preterm birth
GDM: Gestational Diatetes Mellitus
SGA: Small for gestational age
DID: Difference-in-differences
