# Supplementary material for: Estimating the effect of timing of earned income tax credit refunds on perinatal outcomes: a quasi-experimental study of California births
Source: BMC Public Health. 2023 Nov 7;23:2180. doi: 10.1186/s12889-023-16920-0 (PMC10629068; doi:10.1186/s12889-023-16920-0)
Supplement: Supplementary file 1 — Additional file 1: Supplementary Online Content, Supplementary Tables 1, 2 and 3. [file 12889_2023_16920_MOESM1_ESM.docx]

**Supplementary Online Content**

*Model Equation*

The equation for this model was specified as:

$$Y_{ij}=\beta_{0}+\beta_{1}EITC x Trimester+ \beta_{2}EITC+ \beta_{3}Trimester+ \beta_{4}Covariates+ \varepsilon$$

Here, outcomes *Y* are estimated for infants (*i*) born to mothers(*j*)*.* The coefficient of interest, 𝛽_1_, represents the vector of DID coefficients of interest, which capture the effect of EITC refund timing during trimesters one, two, or three compared with preconception. *Covariates* is a vector of infant and maternal covariates, described above. Standard errors were clustered at the level of the mother to account for correlated observations among multiple births to one woman.

*Secondary analyses*

First, we tested whether results were sensitive to the choice of cutpoint for EITC eligibility: we ran models using more restrictive probabilities of 0.6 and 0.7.

Second, to avoid bias, we truncated the sample to exclude those conception cohorts from March and April 2006, given these would have had less opportunity to be classified as preterm births when the observation period began in 2007. Similarly, we truncated cohorts to exclude births conceived April, May, and June of 2012; for these conception cohorts, only births at early gestational ages would be captured in the data that ends in December 2012. For this truncated analysis, the final sample size was 2,663,467.

**Sensitivity analyses**

Analyses in which EITC eligibility was determined based on a probability threshold of 0.6 and 0.7 yielded similar results to the main analysis (eTable1 and eTable2). Truncating the study population to conception cohorts that had full risk for all gestational age outcomes also demonstrated similar results (eTable3).

**Supplementary Tables**

eTable1: DID coefficients for trimester of EITC receipt on perinatal outcomes with predicted EITC eligibility of p>0.6 from PSID algorithm

|  | Preterm birth | SGA | Preeclampsia or gestational hypertension | Gestational diabetes |
| --- | --- | --- | --- | --- |
| First Trimester | 0.00 | 0.02 | 0.10 | 0.04 |
|  | [-0.21, .21] | [-.18, 0.23] | [-0.07, 0.26] | [-0.18, 0.27] |
| Second Trimester | -0.10 | 0.08 | 0.15 | 0.11 |
|  | [-0.31, 0.11] | [-0.13, 0.39] | [-0.02, 0.31] | [-0.11, 0.34] |
| Third Trimester | -0.55 | 0.24 | 0.02 | 0.17 |
|  | [-0.75, -0.36] | [0.03, 0.45] | [-0.14, 0.19] | [-0.05, 0.40] |

Coefficients represent the interaction term between EITC receipt in the first, second or third trimester compared to preconception. Coefficients for binary outcomes were multiplied by 100 and therefore represent a change in percentage points. Values in Brackets represent 95% confidence intervals. Analyses involved multivariable linear models (i.e., linear probability models for binary outcomes) with robust standard errors clustered by mother. Covariates included mother’s race/ethnicity, education, insurance, age, parity and infant’s sex and year of birth.

eTable2: DID coefficients for trimester of EITC receipt on perinatal outcomes with predicted EITC eligibility of p>0.7 from PSID algorithm

|  |  |  |  |  |
| --- | --- | --- | --- | --- |
|  | Preterm birth | SGA | Preeclampsia or gestational hypertension | Gestational diabetes |
| First Trimester | -0.07 | 0.13 | -0.11 | 0.02 |
|  | [-1.1, -0.24] | [-0.27, 0.53] | [-0.43, 0.20] | [-0.04, 0.45] |
| Second Trimester | -0.41 | 0.53 | 0.07 | 0.31 |
|  | [-0.83, 0.02] | [0.13, 0.93] | [-0.24, 0.39] | [-0.12, 0.74] |
| Third Trimester | -1.6 | 0.17 | -0.33 | 0.14 |
|  | [-1.96, -1.16] | [-0.24, 0.58] | [-0.65, -.01] | [-0.29, 0.57] |

Coefficients represent the interaction term between EITC receipt in the first, second or third trimester compared to preconception. Coefficients for binary outcomes were multiplied by 100 and therefore represent a change in percentage points. Values in Brackets represent 95% confidence intervals. Analyses involved multivariable linear models (i.e., linear probability models for binary outcomes) with robust standard errors clustered by mother. Covariates included mother’s race/ethnicity, education, insurance, age, parity and infant’s sex and year of birth.

eTable3: DID coefficients for trimester of EITC receipt on perinatal outcomes with truncated sample

|  | Preterm birth | SGA | Preeclampsia or gestational hypertension | Gestational diabetes |
| --- | --- | --- | --- | --- |
|  |  |  |  |  |
| First Trimester | -0.20 | 0.06 | 0.12 | 0.00 |
|  | [-0.39, 0.01] | [-0.13 , 0.26] | [-0.03, 0.28] | [-0.19, 0.20] |
| Second Trimester | -0.23 | 0.29 | 0.25 | 0.08 |
|  | [-0.42, -0.04] | [0.10, 0.48] | [0.09, 0.41] | [-0.11, 0.27] |
| Third Trimester | -0.53 | 0.38 | 0.17 | 0.09 |
|  | [-0.71, -0.36] | [0.20, 0.58] | [0.01, 0.33] | [-0.10, 0.29] |

Coefficients represent the interaction term between EITC receipt in the first, second or third trimester compared to preconception. Coefficients for binary outcomes were multiplied by 100 and therefore represent a change in percentage points. Values in Brackets represent 95% confidence intervals. Analyses involved multivariable linear models (i.e., linear probability models for binary outcomes) with robust standard errors clustered by mother. Covariates included mother’s race/ethnicity, education, insurance, age, parity and infant’s sex and year of birth.
